# LONELINESS FOLLOWING WIDOWHOOD: THE ROLE OF THE MILITARY AND SOCIAL SUPPORT

**DOI:** 10.1093/geroni/igz038.2090

**Published:** 2019-11-08

**Authors:** Brittany M King, Dawn Carr, Miles G Taylor

**Affiliations:** Florida State University, Tallahassee, Florida, United States

## Abstract

Social support provides important benefits following widowhood. One context promoting social support throughout life may be the military, where benefits extend to both service members and their spouses. A substantial proportion of older men served in the military, so many widowed women today were married to veterans. We tested two hypotheses: 1) surviving military spouses will experience lower persistent loneliness following widowhood compared to their nonmilitary counterparts, and 2) this benefit is explained by increased emotional and structural social support. Our study uses the Health and Retirement Study (HRS) to examine changes in loneliness following widowhood among spouses of veterans and nonveterans. We used OLS regression and mediation tests to address our hypotheses. Overall, results supported our hypotheses. Widows of veterans reported lower levels of loneliness following widowhood compared to nonveteran widows (=-0.122; p<0.05). Emotional and structural social support mediated the relationship between veteran status of the deceased spouse and loneliness. Specifically, the beneficial effect of veteran status was reduced by almost 50% and became nonsignificant. Our findings suggest the military may facilitate lifelong cultivation of social support that flows not only to veterans but also to their families. These findings are suggest that the military may offer important opportunities to cultivate emotional and structural social supports that enhance the ability of veteran wives to more readily adjust to widowhood. Additionally, they emphasize the importance of having social support in later life when faced with adversity, as it seems to ameliorate some of the negative effects.

